# Differential Transcriptomic Landscapes of SARS-CoV-2 Variants in Multiple Organs from Infected Rhesus Macaques

**DOI:** 10.1016/j.gpb.2023.06.002

**Published:** 2023-07-13

**Authors:** Tingfu Du, Chunchun Gao, Shuaiyao Lu, Qianlan Liu, Yun Yang, Wenhai Yu, Wenjie Li, Yong Qiao Sun, Cong Tang, Junbin Wang, Jiahong Gao, Yong Zhang, Fangyu Luo, Ying Yang, Yun-Gui Yang, Xiaozhong Peng

**Affiliations:** 1 National Kunming High-level Biosafety Primate Research Center, Institute of Medical Biology, Chinese Academy of Medical Sciences and Peking Union Medical College, Kunming 650118, China; 2 State Key Laboratory of Medical Molecular Biology, Department of Molecular Biology and Biochemistry, Institute of Basic Medical Sciences, Medical Primate Research Center, Neuroscience Center, Chinese Academy of Medical Sciences, School of Basic Medicine, Peking Union Medical College, Beijing 100005, China; 3 CAS Key Laboratory of Genomic and Precision Medicine, Collaborative Innovation Center of Genetics and Development, College of Future Technology, Beijing Institute of Genomics, Chinese Academy of Sciences and China National Center for Bioinformation, Beijing 100101, China; 4 Sino-Danish College, University of Chinese Academy of Sciences, Beijing 100049, China; 5 Institute of Stem Cell and Regeneration, Chinese Academy of Sciences, Beijing 100101, China; 6 Institute of Laboratory Animal Science, Chinese Academy of Medical Sciences and Peking Union Medical College, Beijing 100021, China

**Keywords:** SARS-CoV-2, Variant of concern, Subgenomic RNA, Rhesus macaque, Transcriptome

## Abstract

Severe acute respiratory syndrome coronavirus 2 (**SARS-CoV-2**) caused the persistent coronavirus disease 2019 (COVID-19) pandemic, which has resulted in millions of deaths worldwide and brought an enormous public health and global economic burden. The recurring global wave of infections has been exacerbated by growing variants of SARS-CoV-2. In this study, the virological characteristics of the original SARS-CoV-2 strain and its **variants of concern** (VOCs; including Alpha, Beta, and Delta) *in vitro*, as well as differential transcriptomic landscapes in multiple organs (lung, right ventricle, blood, cerebral cortex, and cerebellum) from the infected **rhesus macaques**, were elucidated. The original strain of SARS-CoV-2 caused a stronger innate immune response in host cells, and its VOCs markedly increased the levels of **subgenomic RNAs**, such as *N*, *Orf9b*, *Orf6*, and *Orf7ab*, which are known as the innate immune antagonists and the inhibitors of antiviral factors. Intriguingly, the original SARS-CoV-2 strain and Alpha variant induced larger alteration of RNA abundance in tissues of rhesus monkeys than Beta and Delta variants did. Moreover, a hyperinflammatory state and active immune response were shown in the right ventricles of rhesus monkeys by the up-regulation of inflammation- and immune-related RNAs. Furthermore, peripheral blood may mediate signaling transmission among tissues to coordinate the molecular changes in the infected individuals. Collectively, these data provide insights into the pathogenesis of COVID-19 at the early stage of infection by the original SARS-CoV-2 strain and its VOCs.

## Introduction

Severe acute respiratory syndrome coronavirus 2 (SARS-CoV-2) caused the coronavirus disease 2019 (COVID-19) pandemic [1], [2], which has been posing a great threat to global public health and is accompanied by the evolution of the virus genome [3], [4]. Since the emergence of SARS-CoV-2, its genome has been under constant mutation to adapt to the host system, and several variants of concern (VOCs) have emerged and become prevalent across the world [5], [6], leading to the improvement of survival advantages, such as higher transmissibility, greater receptor affinity, viral replication, and immune escape [7]. The characteristics and differences of SARS-CoV-2 original strain and its VOCs remain to be further explored.

Considering the differential molecular characteristics of variations, we intended to explore the pathogenesis of SARS-CoV-2 original strain (GD108) and its VOCs (including Alpha, Beta, and Delta) both *in vitro* and *in vivo*. The lineage Alpha (B.1.1.7) was reported in the United Kingdom; the lineage Beta (B.1.351) was identified in South Africa; and the lineage Delta (B.1.617.2) was first discovered in India [8], [9], [10], [11]. The mutations of VOCs have mainly occurred in the receptor binding domain (RBD) and the N-terminal domain of the Spike (S) protein [12]. S protein, which appears on the surface of viruses, is recognized by the host cell receptor, such as angiotensin-converting enzyme 2 (ACE2), which promotes the entry of the virus into the cell [13]. The mutations present in the genome of SARS-CoV-2 variants have a significant effect on the biological and immunogenic characteristics of the virus, which is strongly associated with transmissibility and immunological response in humans [14]. SARS-CoV-2 infection causes pneumonia syndrome in the lung as one of the respiratory viruses [15]. Moreover, the virus also affects multiple organs, such as liver [16], kidney [17], heart [18], vascular system [19], and nervous system [20]. The molecular changes of multiple organs caused by the original SARS-CoV-2 have been illustrated in a previous study [21]. However, the effects of different SARS-CoV-2 VOCs on the transcriptomes of different tissues remain unknown.

In this study, we adopted the whole-transcriptome sequencing to establish the transcriptome-wide molecular changes of SARS-CoV-2 original strain and its VOCs in both MA104 cells (monkey embryonic kidney cells) at 48 h post-infection (hpi) and rhesus macaques’ tissues, including cerebral cortex, cerebellum, right ventricle, and peripheral blood, at 5 days post-infection (dpi). We revealed that SARS-CoV-2 original strain induced a stronger immune response and VOCs markedly increased the expression of subgenomic RNAs (sgRNAs) *in vitro*, and SARS-CoV-2 original strain and its VOCs induced RNA dysregulation in rhesus macaques’ multiple tissues. These findings improve the understanding of the pathogenesis of SARS-CoV-2 original strain and its VOCs in the infected individuals.

## Results

### SARS-CoV-2 original strain induces a stronger innate immune response

**Figure 1:**
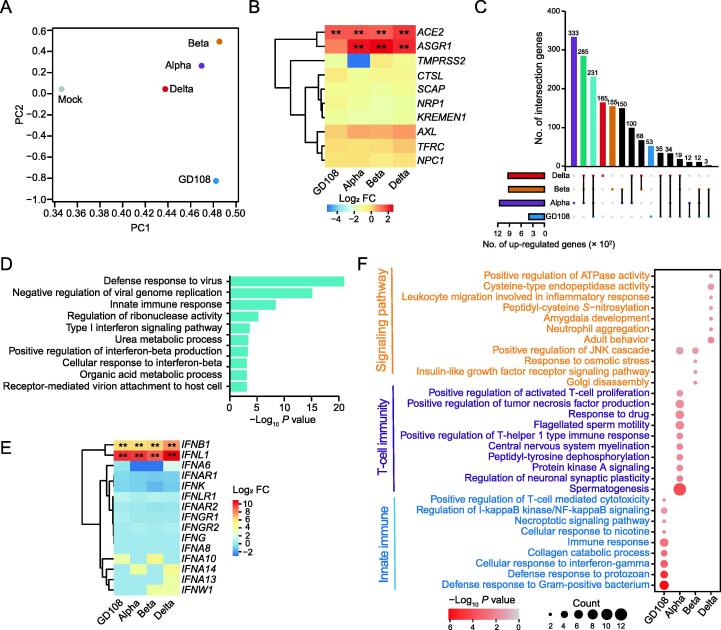
**Infection of SARS-CoV-2 original strainand its VOCs induces innate immune response in host cells** **A.** PCA of the whole transcriptomes of MA104 cells separately infected by SARS-CoV-2 orinial stain (GD108) and its VOCs (Alpha, Beta, and Delta) for 48 h. Mock indicates MA104 cells treated with PBS for 48 h. **B.** Heatmap showing the RNA expression changes of SARS-CoV-2 receptor genes after SARS-CoV-2 infection. The asterisk represents dysregulated RNAs with statistical significance (*, FDR < 0.05; **, FDR < 0.01). **C.** Upset plot displaying the overlap of the up-regulated RNAs after SARS-CoV-2 infection. **D.** Bar plot showing the enriched functional annotation of the commonly up-regulated RNAs after SARS-CoV-2 VOC infection as shown in (C). **E.** Heatmap displaying the RNA expression changes of IFNs after SARS-CoV-2 infection. The asterisk represents dysregulated RNAs with statistical significance (*, FDR < 0.05; **, FDR < 0.01). **F.** Bubble chart showing the enrichment of annotated GO terms by the specifically up-regulated RNAs after infection with SARS-CoV-2 original strain and its VOCs. SARS-CoV-2, severe acute respiratory syndrome coronavirus 2; VOC, variant of concern; PBS, phosphate buffer saline; PCA, principal component analysis; PC, principal component; FC, fold change; GO, Gene Ontology; IFN, interferon; FDR, false discovery rate.

**Supplementary Figure S1:**
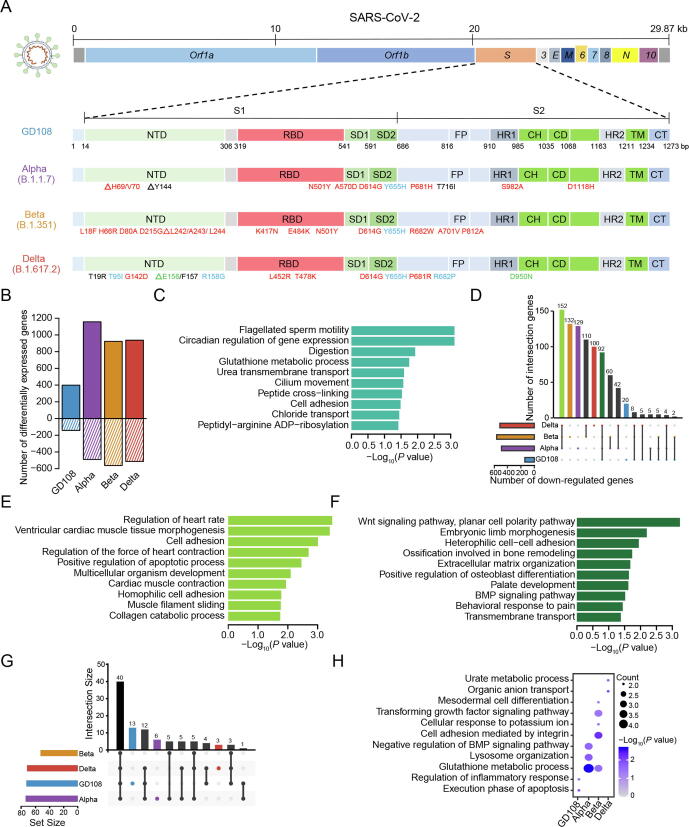
**SARS-CoV-2 VOCsinduce greater transcriptional dysregulation A.** Top: schematic depiction of the SARS-CoV-2 genomic arrangement and proteins. Bottom: outline of the specific amino acid exchanges of S protein in three VOCs Alpha (B.1.1.7), Beta (B.1.351), and Delta (B.1.617.2) compared with the reference original strain (GD108) sequence. The comparison was performed by the first-generation sequencing. **B.** Bar plot displaying the number of dysregulated RNAs after SARS-CoV-2 infection. The positive bars represent the number of up-regulated RNAs, while the negative ones are the number of down-regulated RNAs. **C.** Bar plot showing the enrichment of annotated GO terms by the commonly up-regulated RNAs after SARS-CoV-2 VOC infection. **D.** Upset plot depicting the overlap of the down-regulated RNAs after SARS-CoV-2 infection. **E.** Bar plot showing the enrichment of annotated GO terms by the commonly down-regulated RNAs in the SARS-CoV-2 VOC-infected cells. **F.** Bar plot showing the enrichment of annotated GO terms by the commonly down-regulated RNAs in cells infected by SARS-CoV-2 original strain and its VOCs **G.** Upset plot depicting the overlap of the up-regulated ISGs after SARS-CoV-2 infection. **H.** Bubble chart displaying the enrichment of annotated GO terms by the specifically down-regulated RNAs after infection by SARS-CoV-2 original strain and its VOCs. IFN, interferon; ISG, IFN-stimulated gene.

To investigate the molecular alteration of host cells after infection, we carried out whole-transcriptome sequencing on MA104 cells respectively infected by SARS-CoV-2 original strain (GD108) and its VOCs (Alpha, Beta, and Delta) for 48 h, and cells treated with phosphate buffer saline (PBS; mock) were used as a control (Figure S1A). We found that the VOCs induced more similar transcriptional expression characteristics compared to GD108 (Figure 1A; Table S1). ACE2 was identified as the main receptor of SARS-CoV-2 [22], and its RNA level was obviously elevated in the GD108- and VOC-infected cells compared to the mock-treated cells (Figure 1B). Intriguingly, the RNA level of asialoglycoprotein receptor-1 (ASGR1; the S protein-binding partner [23]) was substantially increased in VOC-infected cells, which might have further promoted higher proportions of transcription changes compared with GD108-infected cells (Figure S1B; Table S2). Moreover, the VOCs, but not GD108, commonly up-regulated the expression of 285 RNAs involved in the regulation of gene expression and metabolic pathways (Figure 1C, Figure S1C), and down-regulated the expression of 152 RNAs that participate in cardiac muscle morphogenesis and contraction (Figure S1D and E) in the infected cells. The cell–cell adhesion-realated and signaling pathway-related RNAs were commonly down-regulated after infection by all SARS-CoV-2 strains (Figure S1D and F), while the commonly up-regulated RNAs induced by all SARS-CoV-2 strains were significantly related to the pathways of antiviral response and innate immunity (Figure 1C and D), which are the first line of defense against the virus [24]. Furthermore, the RNA levels of type I interferons (IFNs) with antiviral effect [25] were induced by SARS-CoV-2 infection (Figure 1E). The expression of IFN-stimulated genes (ISGs) was also commonly induced after SASR-CoV-2 infection (Figure S1G). Intriguingly, the specifically up-regulated RNAs (*n* = 53) after GD108 infection were annotated in innate immune and inflammatory pathways; Alpha infection specifically induced the up-regulation of RNAs (*n* = 333) related to T-cell immunity; and signaling pathway-related RNAs were specifically up-regulated after Beta and Delta infection (*n* = 155 and 165, respectively) (Figure 1C and F). The specifically down-regulated RNAs were involved in metabolic and signaling pathways (Figure S1H). Collectively, although the VOC infection disturbed the transcription of host cells to a greater extent, the original strain of SARS-CoV-2 induced stronger innate immune and inflammatory responses.

### SARS-CoV-2 VOCs display enhanced sgRNA expression of innate immune antagonists in the infected MA104 cells

**Figure 2:**
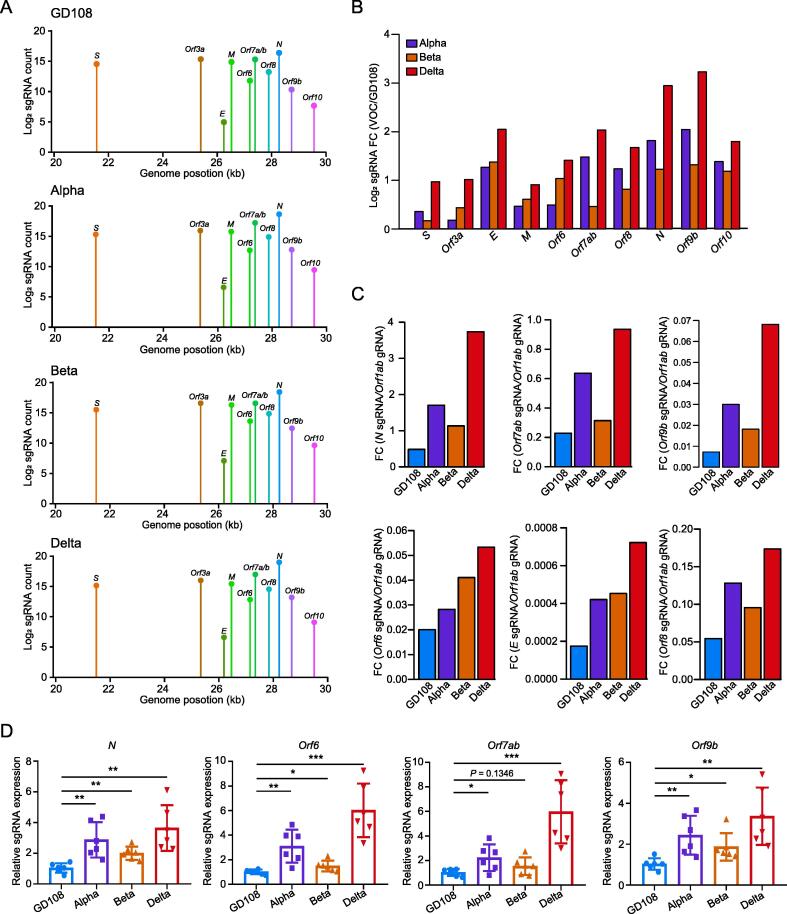
**SARS-CoV-2 VOCs produce more highly abundant sgRNAs of innate immune antagonists** **A.** Log_2_-normalized sgRNA counts (dot height) projected onto their identified start sites on the SARS-CoV-2 genome (48 hpi). Canonical sgRNAs and the non-canonical *Orf9b* sgRNA are depicted. **B.** Bar plot showing the log_2_ sgRNA FC of VOCs to GD108 normalized to total gRNA of RNA-seq. **C.** Quantification of the sgRNA levels of *N*, *Orf7ab*, *Orf9b*, *Orf6*, *E*, and *Orf8* compared with the gRNA level of *Orf1ab* by RNA-seq. **D.** Assessment of sgRNA abundance (*N*, *Orf6*, *Orf9b*, and *Orf7ab*) by qRT-PCR (*n* = 6; *, *P* < 0.05; **, *P* < 0.01; ***, *P* < 0.001). The error bars represent the SD. sgRNA, subgenomic RNA; hpi, hours post-infection; RNA-seq, RNA sequencing; gRNA, genomic RNA; SD, standard deviation.

**Supplementary Figure S2:**
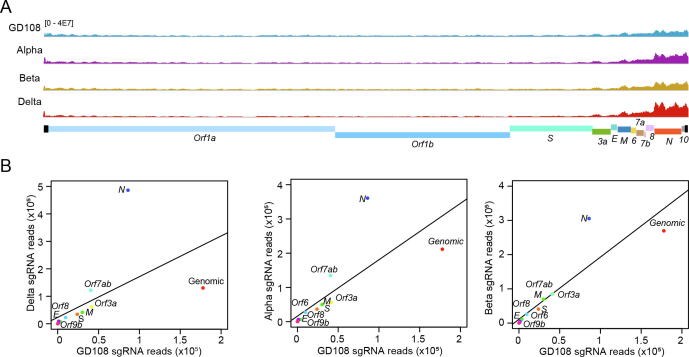
**Genomictracksand sgRNA abundance of SARS-CoV-2VOCs A.** IGV displaying the genome tracks of SARS-CoV-2 VOCs in MA104 cells. **B.** Scatter plot of sgRNA abundance in Alpha, Beta, and Delta compared with GD108. IGV, Integrative Genomics Viewer.

To uncover the differences among VOCs and GD108 that underlie the differential host responses, we further examined the viral RNA sequencing (RNA-seq) data in the infected MA104 cells at 48 hpi. Expectedly, we observed that VOCs showed higher RNA abundance than GD108 did (Figure S2A). We further evaluated the sgRNA levels by selecting transcripts with the 5′-leader sequence during sgRNA synthesis (see Materials and methods) and found that the sgRNAs of nucleocapsid (N) protein, Orf9b, and Orf6, defined as immune antagonists, displayed markedly higher RNA abundance in VOC-infected cells than in GD108-infected cells (Figure 2A; Table S3). The up-regulation of immune antagonists such as N, Orf9b, and Orf6 in Alpha has been reported to enhance immune evasion and viral transmission [26]. Notably, we compared the sgRNA levels of VOCs with those of GD108, and found that Orf7ab, envelope (E) protein, and Orf8 also displayed substantially increased sgRNA abundance, especially in Delta strain (Figure 2B, Figure S2B). We also observed higher sgRNA abundance relative to the genomic RNA (gRNA) of *Orf1ab* in Delta and Alpha strains than in GD108 (Figure 2C). We further confirmed the up-regulated expression of *N*, *Orf9b*, *Orf6*, and *Orf7ab* in VOCs using qRT-PCR, especially in Alpha and Delta strains (Figure 2D). Collectively, these results suggest that the sgRNA synthesis of VOCs is substantially increased in the host cells, which may enhance the VOCs immune evasion and rapid transmission.

### SARS-CoV-2 original and Alpha strains significantly disturb RNA expression in the infected rhesus macaques’ tissues

**Figure 3:**
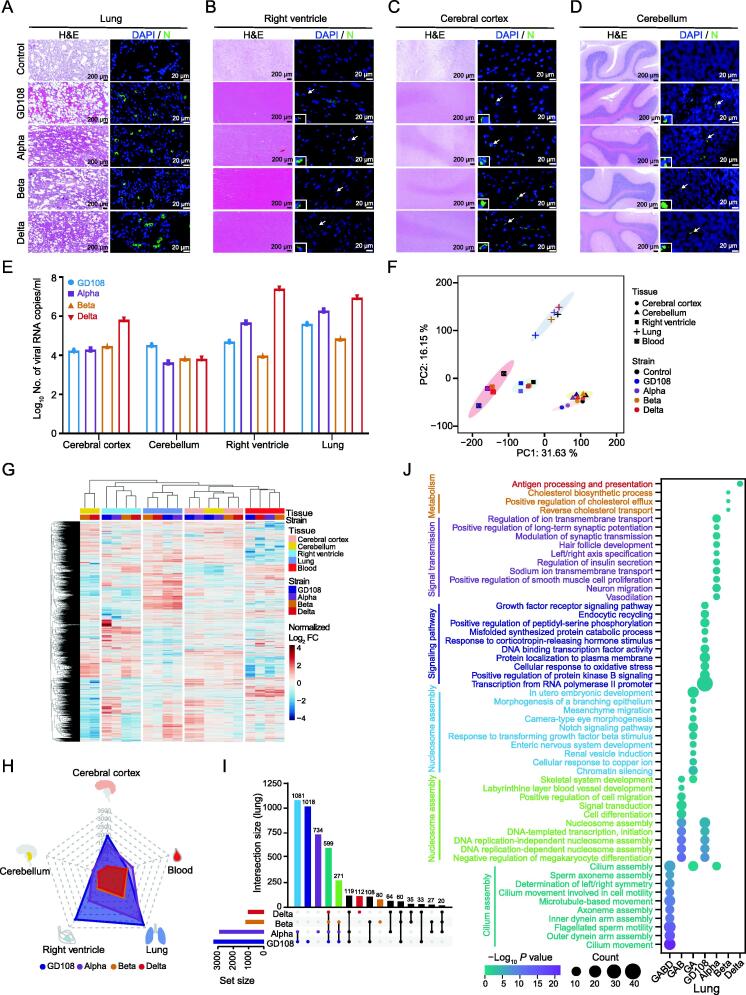
**Disordered transcription induced by the infection of SARS-CoV-2 original strain and its VOCs in rhesus macaques** H&E and immunofluorescence staining of N protein (green) in lung (**A**),  right ventricle (**B**), cerebral cortex (**C**), and cerebellum (**D**) at 5 dpi after SARS-CoV-2 infection. The uninfected macaque tissues were used as controls. Scale bar, 200 or 20 μm, as indicated. **E.** qRT-PCR for viral RNA loads in the aforementioned four tissues at 5 dpi after SARS-CoV-2 infection. **F.** PCA displaying the similarity of whole-transcriptome expression patterns for each type of tissues with or without SARS-CoV-2 infection. **G.** Heatmap showing the FCs of differentially expressed genes in tissues after SARS-CoV-2 infection at 5 dpi. **H.** Radar plot displaying the number of significantly up-regulated RNAs in tissues after SARS-CoV-2 infection. Different colors represent different SARS-CoV-2 strains. **I.** Upset plot depicting the overlap of the up-regulated RNAs in lung tissue after SARS-CoV-2 infection at 5 dpi. **J.** Bubble chart displaying the enrichment of annotated GO terms by the RNA sets defined in (I). H&E, Hematoxylin and Eosin; dpi, days post-infection; DAPI, 4′,6-diamidino-2-phenylindole; GA, GD108 and Alpha; GAB, GD108, Alpha, and Beta; GABD, GD108, Alpha, Beta, and Delta.

**Supplementary Figure S3:**
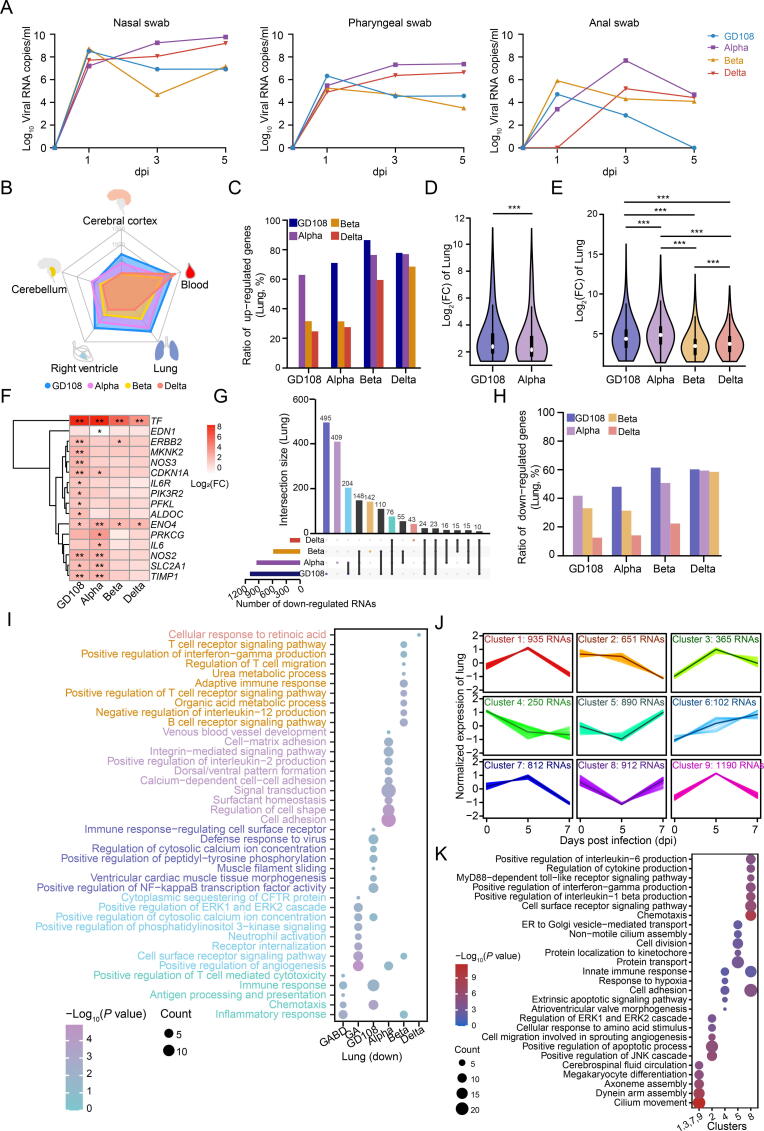
**Infection ofSARS-CoV-2original strainand its VOCscommonly suppresses the immune response of lung tissues at 5 dpi A.** Line plot showing viral RNA loads in nasal, pharyngeal, and anal swabs. **B.** Radar plot displaying the number of the significantly down-regulated RNAs in tissues after SARS-CoV-2 infection. The different colors represent different SARS-CoV-2 strains. **C.** Bar plot displaying the proportion of the commonly up-regulated RNAs in the infected lung tissues between every two SARS-CoV-2 strains. **D.** Violin plot showing the expression FCs of the commonly up-regulated RNAs (1081 RNAs, Figure 3I) in lung tissues after SARS-CoV-2 GD108 and Alpha infections. **E.** Violin plot showing the expression FCs of the commonly up-regulated RNAs (599 RNAs, Figure 3I) in lung tissues after infection of SARS-CoV-2 original strain and its VOCs. **F.** Heatmap displaying the expression FCs of the dysregulated RNAs involved in HIF-1 signaling pathway in lung tissues after SARS-CoV-2 infection. **G.** Upset plot depicting the overlap of the down-regulated RNAs in lung tissues after SARS-CoV-2 infection at 5 dpi. **H.** Bar plot displaying the proportion of the commonly down-regulated RNAs in the infected lung tissues between every two SARS-CoV-2 strains. **I.** Bubble chart showing enrichment of the annotated GO terms by the RNA sets defined in (G). **J.***K*-means clustering for the normalized expression of the dysregulated RNAs after the infection with the original strain of SARS-CoV-2 in lung tissues for 5 and 7 days. **K.** Bubble chart showing the enriched GO terms by the RNA clusters from (H).

Rhesus monkeys were infected with SARS-CoV-2 original stain and its VOCs at 1 × 10^7^ plaque-forming unit (PFU), and the tissues were collected at 5 dpi. Body weight and body temperature did not change significantly in rhesus monkeys during the study (Table S4). Compared with the uninfected macaques, Hematoxylin and Eosin (H&E) staining results showed that SARS-CoV-2 induced severe interstitial pneumonia with thickened alveolar septa, edema, and interstitial congestion; severe infiltration of inflammatory immune cells; type II pneumocyte hyperplasia; and reactive endothelial cells in blood vessels (Figure 3A). The ventricular pathological features of the infected animals included local mild congestion of the heart vessels, scattered yellow-brown pigmentation, and thrombus formation in the coronary vascular lumen (Figure 3B). The cerebral cortex and cerebellum sections of the infected macaques showed relatively less severe pathological signs, where cell proliferation was observed in a small proportion of glial cells in some cortex sections at 5 dpi (Figure 3C and D). Viral loads were determined in nasal, pharyngeal, and anal swabs at 1 dpi, 3 dpi, and 5 dpi, respectively (Figure S3A). The infection of SARS-CoV-2 in the tissues was further validated by immunofluorescence staining (Figure 3A–D) and qRT-PCR (Figure 3E) at 5 dpi.

To further investigate the transcriptomic changes after SARS-CoV-2 infection in the rhesus macaques, we evaluated the transcription using RNA-seq data. We observed significant transcriptional similarity for each tissue and similar transcription patterns of brain tissues (Figure 3F; Table S5). Each tissue showed similar transcriptional changes after the infection with different SARS-CoV-2 strains, and the tissues from brain displayed some co-dysregulated RNAs (Figure 3G). Intriguingly, the infections with GD108 and Alpha strains induced markedly transcriptional changes relative to Beta and Delta strains in the lung, cerebral cortex, cerebellum, and right ventricle tissues, but there were no significant differences in the blood (Figure 3H, Figure S3B; Table S6). Our results suggest that GD108 and Alpha infections significantly disturb the transcription in tissues, while Delta strain causes slight transcription changes with the highest viral loads, and Beta strain has relatively weak infectivity and disturbance.

### Immune response is suppressed in the infected lung tissues

As a respiratory virus, SARS-CoV-2 primarily causes pneumonia and immunopathology in lung tissues [27]. We next evaluated the transcriptional changes of lung tissues infected by SARS-CoV-2 original strain and its VOCs, and found that GD108 and Alpha infections significantly induced the up-regulation of RNA transcription (Figure 3I), and more than 60% of the up-regulated RNAs induced by one strain were also up-regulated in the lung tissues infected by other strains (Figure S3C). We also observed higher transcriptional changes of the commonly up-regulated RNAs in GD108-infected and Alpha-infected lungs than those in the lung tissues infected by the other two strains (Figure S3D), and these were involved in cilium assembly pathways (Figure 3J). Moreover, GD108 infection induced significantly greater transcriptional changes than Alpha (Figure S3E), and these were involved in cellular response, morphogenesis, and signaling pathways (Figure 3J). In addition, GD108 infection specifically induced up-regulation of oxidative stress-related RNAs (Figure 3J). Considering the signs of hypoxia after SARS-CoV-2 infection [28], the dysregulated RNAs were evaluated in hypoxia-inducible factor-1 (HIF-1) pathways derived from Kyoto Encyclopedia of Genes and Genomes (KEGG) [29], and we observed that these factors were significantly induced in GD108-infected lungs compared with the control group (Figure S3F). In addition, we further evaluated the down-regulated RNAs in the infected lung tissues (Figure S3G), and found that there were less than 60% commonly down-regulated RNAs among different strain-infected lungs (Figure S3H), while immune and inflammatory responses were commonly suppressed at 5 dpi (Figure S3I). In other words, these results showed that the original SARS-CoV-2 strain infection significantly disturbed the transcription in lung tissues. We had previously illustrated the transcriptional changes of lung tissues infected by the original strain at 7 dpi [21], so we combined dysregulated RNAs after SARS-CoV-2 infection for 5 and 7 days in lung tissues to characterize the transcriptional changes of RNA clusters over time (Figure S3J). Of note, we observed that immune response-related RNAs were specifically suppressed at 5 dpi and then remained stable or increased at 7 dpi in Clusters 4, 5, and 8 (Figure S3K). To sum up, immune response is specifically suppressed in the lung tissues infected by SARS-CoV-2 origianl strain and its VOCs at 5 dpi.

### Cerebral cortex is more susceptible than cerebellum upon SARS-CoV-2 infection

**Figure 4:**
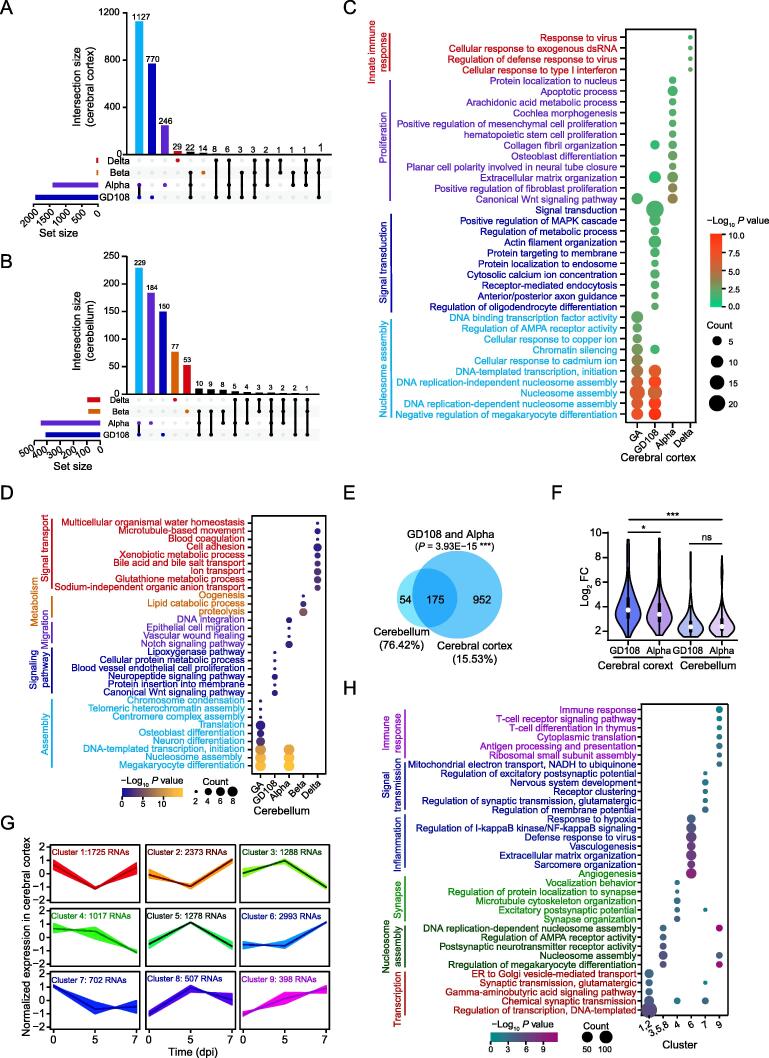
**SARS-CoV-2 original and Alpha strains induce greater transcriptional changes in cerebral cortex than in cerebellum** **A.** Upset plot showing the intersection of the significantly up-regulated RNAs in cerebral cortex after SARS-CoV-2 infection. **B.** Upset plot showing the intersection of the significantly up-regulated RNAs in cerebellum after SARS-CoV-2 infection. **C.** Bubble chart displaying the enriched GO terms for the different RNA sets defined in (A). **D.** Bubble chart displaying the enriched GO terms for the different RNA sets defined in (B). **E.** Venn plot showing the overlap of the commonly up-regulated RNAs induced in GD108-infected and Alpha-infected cerebral cortex (1127 RNAs) and cerebellum (229 RNAs). **F.** Violin plot displaying the enrichment of the commonly up-regulated RNAs (175 RNAs in E) in cerebral cortex and cerebellum after GD108 and Alpha infection. **G.***K*-means clustering for the normalized expression of the dysregulated RNAs in cerebral cortex at 5 and 7 dpi after the SARS-CoV-2 original strain infection. **H.** Bubble chart showing the enriched GO terms by the RNA clusters shown in (G). ns, not significant.

**Supplementary Figure S4:**
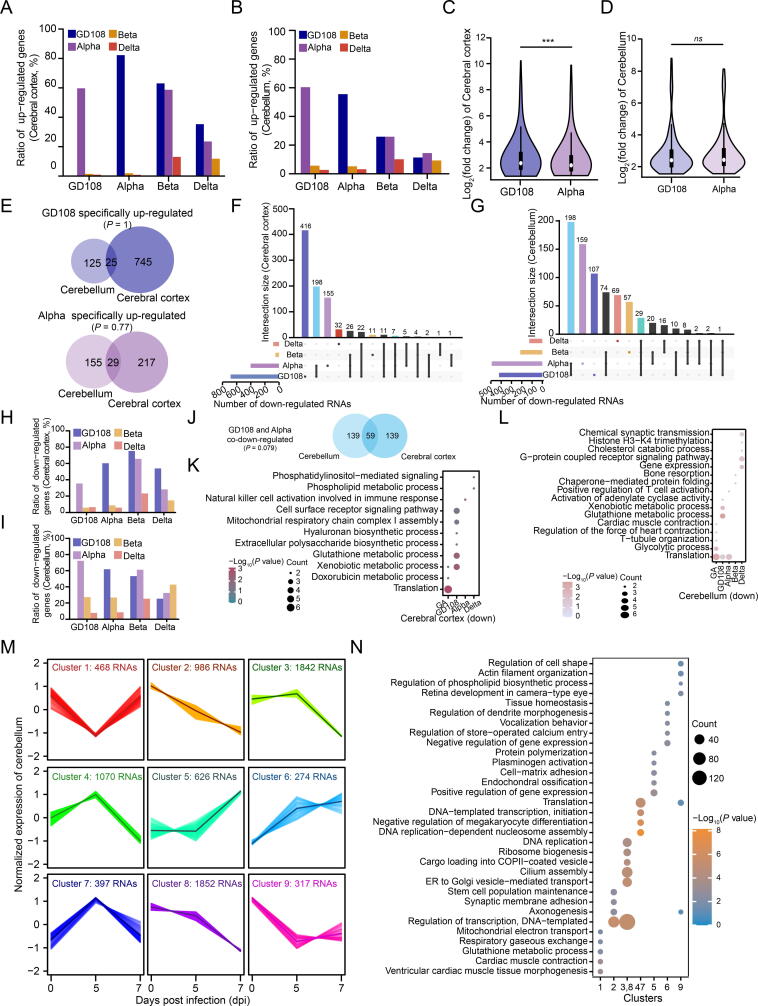
**The transcription of brain tissues is disturbed by SARS-CoV-2original strainand its VOCsA.** Bar plot showing the proportion of the commonly up-regulated RNAs in the infected cerebral cortex between every two SARS-CoV-2 strains. **B.** Bar plot showing the proportion of the commonly up-regulated RNAs in the infected cerebellum between every two SARS-CoV-2 strains. **C.** Violin plot displaying expression FCs of the commonly up-regulated RNAs in cerebral cortex after GD108 and Alpha infections. **D.** Violin plot displaying the exprssion FCs of the commomly up-regulated RNAs in cerebellum after GD108 and Alpha infection. **E.** Venn plot showing the intersection of the up-regulated RNAs in cerebral cortex and cerebellum at 5 dpi after GD108 and Alpha infections. **F.** The intersection of the down-regulated RNAs in cerebral cortex at 5 dpi after SARS-CoV-2 infection. **G.** The intersection of the down-regulated RNAs in cerebellum at 5 dpi after SARS-CoV-2 infection. **H.** Bar plot showing the proportion of the commonly down-regulated RNAs in the infected cerebral cortex between every two SARS-CoV-2 strains. **I.** Bar plot showing the proportion of the commonly down-regulated RNAs in the infected cerebellum between every two SARS-CoV-2 strains. **J.** The intersection of the down-regulated RNAs in cerebral cortex and cerebellum at 5 dpi. **K.** Bubble chart displaying the annotated GO terms for the cerebral cortex RNA sets defined in (J). **L.** Bubble chart displaying the annotated GO terms for the cerebellum RNA sets defined in (J). **M.***K*-means clustering for the normalized expression of the dysregulated RNAs after infection with the original strain of SARS-CoV-2 in cerebellum for 5 and 7 days. **N.** Bubble chart showing the enriched GO terms by the RNA clusters from (L).

We next investigated the transcriptional changes of brain tissues after infection with different SARS-CoV-2 strains. We observed that GD108 and Alpha induced a greater proportion of up-regulated RNAs (Figure 4A and B), and more than 50% of these were commonly up-regulated (Figure S4A and B). However, the commonly up-regulated RNAs showed significantly higher enrichment in GD108-infected than in Alpha-infected cerebral cortex, while there was no substantial difference in the infected cerebellum (Figure S4C and D). Notably, the commonly up-regulated RNAs in GD108-infected and Alpha-infected cerebral cortex and cerebellum were significantly enriched in nucleosome assembly pathways (Figure 4C and D), suggesting similar responses of the brain tissues after SARS-CoV-2 infection. Then, we observed a significant overlap of the commonly up-regulated RNAs in GD108-infected and Alpha-infected cerebral cortex and cerebellum (Figure 4E), but higher enrichment and more up-regulated RNAs in cerebral cortex (Figure 4F), suggesting that GD108 and Alpha infections induce higher proportion of transcriptional changes in cerebral cortex. Of note, we did not observe similar transcriptional changes for GD108 and Alpha specifically induced RNAs in cerebral cortex and cerebellum (Figure S4E). We further evaluated the down-regulated RNAs in cerebral cortex and cerebellum tissues infected with SARS-CoV-2 original strain and its VOCs, and also observed a higher number of down-regulated RNAs in GD108-infected and Alpha-infected tissues (Figure S4F and G), with 40%–60% of common RNAs in cerebral cortex (Figure S4H) and more than 60% of common RNAs in cerebellum (Figure S4I). There was no obvious intersection of the commonly down-regulated RNAs in GD108-infected and Alpha-infected cerebral cortex and cerebellum (Figure S4J), and metabolic and cardiac contraction pathways were enriched by commonly down-regulated RNAs in GD108-infected and Alpha-infected cerebral cortex (Figure S4K) and cerebellum (Figure S4L), respectively. Intriguingly, Alpha infection specifically suppressed immune response-related RNAs in cerebral cortex and cerebellum at 5 dpi (Figure S4K and L). Considering the active inflammatory and immune responses in the GD108-infected cerebral cortex at 7 dpi [21], we combined the time-course transcription data of cerebral cortex and cerebellum for clustering (Figure 4G, Figure S4M). We observed that the inflammatory and immune response-related RNAs were specifically up-regulated in cerebral cortex at 7 dpi (Figure 4H), but not in cerebellum tissues (Figure S4N). Collectively, the cerebral cortex is more susceptible than cerebellum to SARS-CoV-2 infection and shows active immune and inflammatory responses after original SARS-CoV-2 strain infection at 7 dpi.

### Immune and inflammatory responses are activated in the infected right ventricle

**Figure 5:**
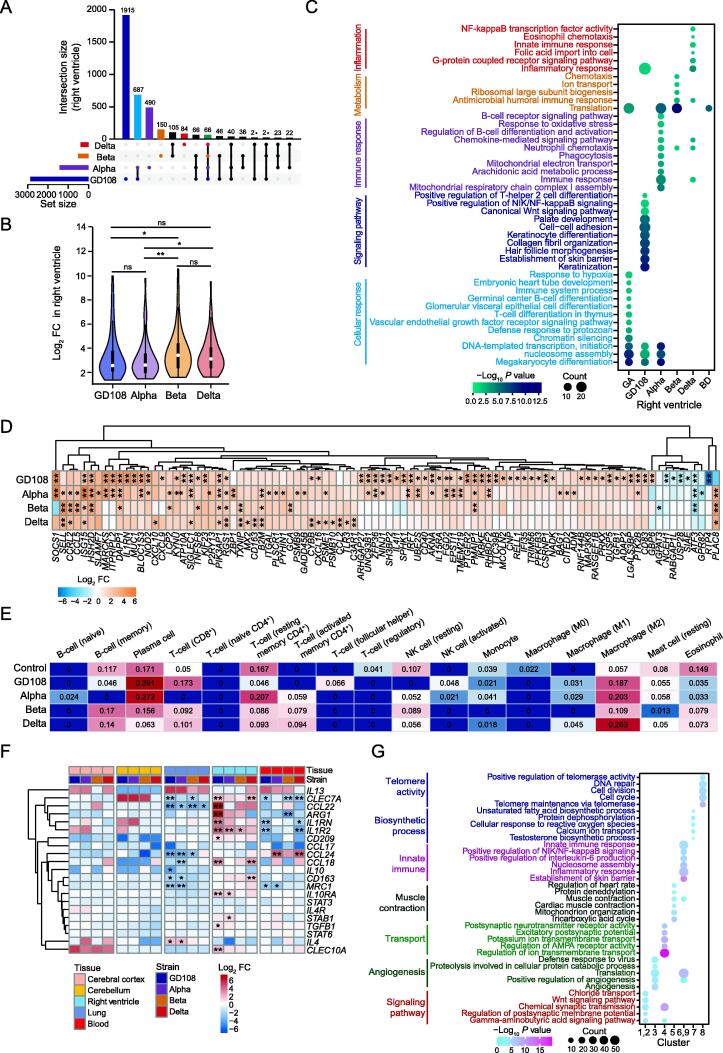
**SARS-COV-2original strainand its VOCsactivate theinnate immune responsein right ventricle** **A.** The intersection of the up-regulated RNAs in the right ventricle after SARS-CoV-2 infection. **B.** Violin plot showing the enrichment of the commonly up-regulated RNAs induced by all SARS-CoV-2 strains in the right ventricle at 5 dpi. **C.** The enriched GO terms for the RNA sets defined in (A). **D.** Heatmap showing the FCs of ISGs in the  right ventricle tissues infected by different SARS-CoV-2 strains. Details of these 94 differentially expressed ISGs are listed in Table S6. *, FDR < 0.05; **, FDR < 0.01. **E.** The predicted proportion of immune cells in the infected right ventricle tissues by the RNA-seq data. **F.** The expression changes of M2 macrophage-induced factors among different infected tissues. *, FDR < 0.05; **, FDR < 0.01. **G.** Bubble chart showing the enriched GO terms for the RNA clusters in Figure S5F. ISG, interferon-stimulated gene; NK, natural killer.

**Supplementary Figure S5:**
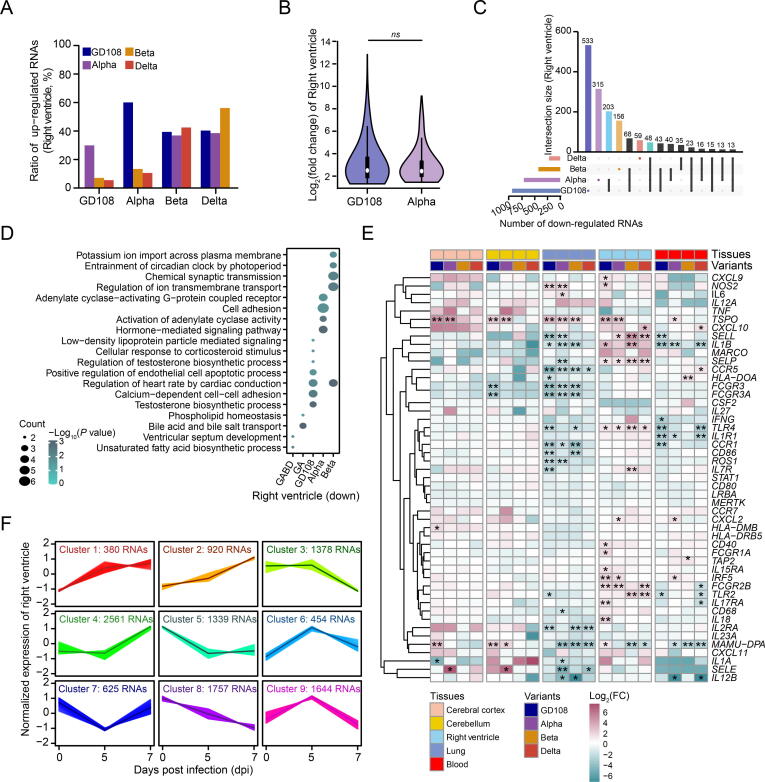
**Transcriptional changes in right ventricle after infection of SARS-CoV-2 origianl strain and its VOCsA.** Bar plot showing the proportion of the commonly up-regulated RNAs in the infected right ventricle tissues between every two SARS-CoV-2 strains. **B.** Violin plot displaying the expression FCs of the commonly up-regulated RNAs in GD108-infected and Alpha-infected right ventricle. **C.** The intersection of the down-regulated RNAs in the right ventricle at 5 dpi. **D.** Bubble chart displaying the enriched GO terms of the RNA sets defined in (C). **E.** Heatmap showing the expression FCs of M1-induced factors among the infected tissues. **F.***K*-means clustering for the normalized expression of RNAs in GD108-infected right ventricle at 5 and 7 dpi.

As the right ventricle tissues showed substantially disturbed transcription (Figure 3H), we further evaluated the intersection of the up-regulated RNAs among different strain infections (Figure 5A) and found that nearly 60% of the up-regulated RNAs induced by Alpha infection were also up-regulated by GD108 infection (Figure S5A). There was no significant difference in the transcriptional changes of the commonly up-regulated RNAs between GD108 and Alpha infections (Figure S5B). Intriguingly, the commonly up-regulated RNAs induced by all SARS-CoV-2 strians showed higher enrichment in Beta and Delta infections (Figure 5B), which was different from the lung and brain tissues. The up-regulated RNA sets were mainly enriched in immune and inflammatory response pathways (Figure 5C), while the down-regulated RNAs were more specific in each strain infection (Figure S5C) and were involved in some basic signaling and development pathways (Figure S5D). Considering the active immune responses in the infected right ventricle, we next evaluated the expression of ISGs and observed significantly increased transcription in the right ventricle infected by GD108 compared to those infected by VOCs (Figure 5D; Table S6). Immune cells were predicted in the right ventricle tissues with or without SARS-CoV-2 infection using CIBERSORT [30], and we found that the proportion of M2 macrophage cells was markedly increased in the infected tissues (Figure 5E). It has been reported that M1 and M2 macrophages are polarized from macrophages and closely related to the inflammatory response [31]. We therefore evaluated the M2-induced and M1-induced factors and found that they were specifically increased with significance in right ventricle post-infection (Figure 5F, Figure S5E). Combining the time-course transcriptome data (Figure S5F), we observed that the specifically increased RNAs at 5 dpi in Cluster 6 and Cluster 9 (Figure S5F) were significantly enriched in active immune and inflammatory response pathways (Figure 5G). Overall, immune- and inflammatory-related RNAs were specifically induced in the right ventricle at 5 dpi.

### Infection with SARS-CoV-2 VOCs induces blood immune response

**Figure 6:**
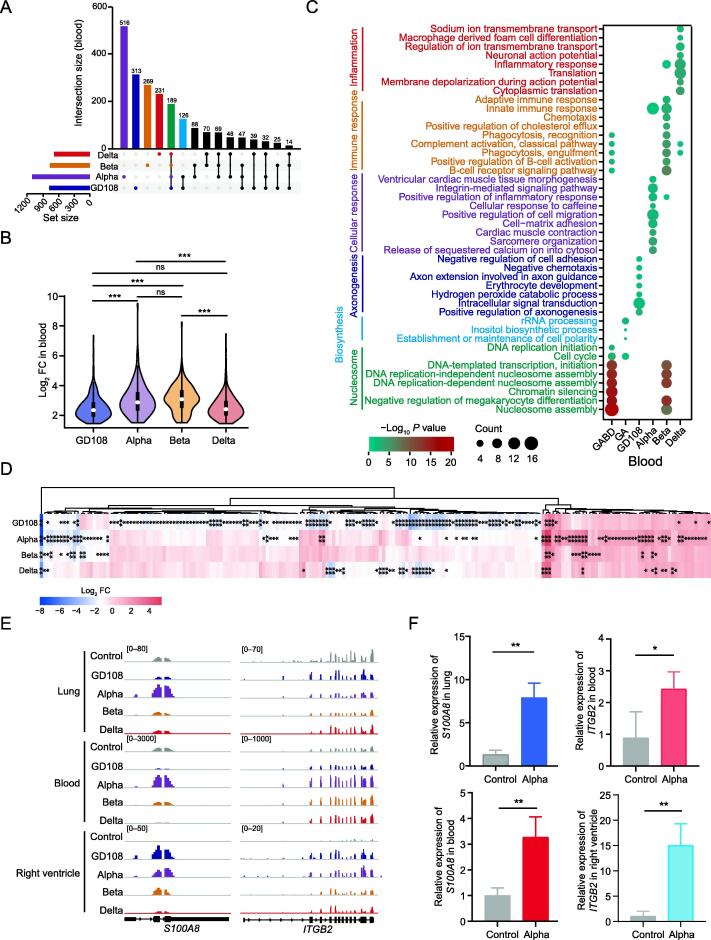
**The immune response in blood is activated by SARS-CoV-2VOCs** **A.** The intersection of the up-regulated RNAs in blood tissues at 5 dpi after SARS-CoV-2 infection. **B.** Violin plot displaying the enrichment of the commonly up-regulated RNAs induced by all SARS-CoV-2 strains in the infected blood tissues. **C.** Bubble chart showing the enriched GO terms for the RNA sets defined in (A). **D.** Heatmap showing the expression changes of ISGs in the infected blood tissues. Details of these 202 differentially expressed ISGs are listed in Table S6. *, FDR < 0.05; **, FDR < 0.01. **E.** Genome browser showing the read abundance along *S100A8* (ligand gene) and *ITGB2* (receptor gene) in lung, blood, and right ventricle from control and infected rhesus macaques. **F.** qRT-PCR analysis showing the up-regulated expression of *S100A8* and *ITGB2* in the Alpha-infected lung/blood and blood/right ventricle tissues, respectively. Three replicates were measured in each tissue sample. *, *P* < 0.05; **, *P* < 0.01 (Student’s *t*-test).

**Supplementary Figure S6:**
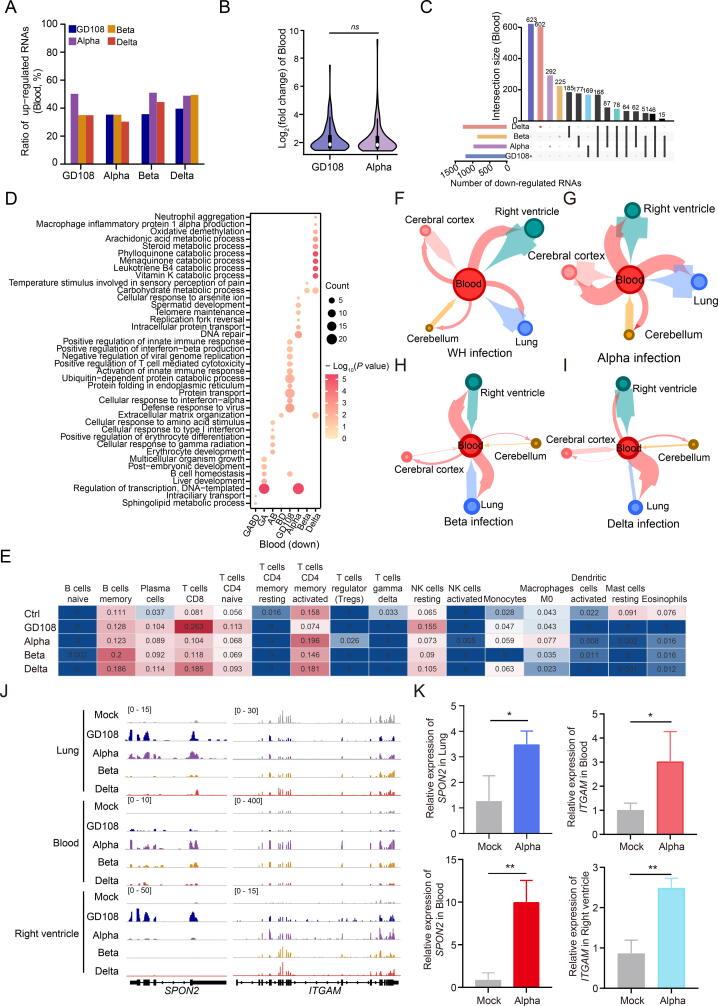
**Transcriptomic changes inblood tissuesafter infection of SARS-CoV-2 origianl strain and its VOCsA.** The proportion of the commonly up-regulated RNAs in the infected blood tissues between every two SARS-CoV-2 strains. **B.** Violin plot showing the transcriptional FCs of the commonly up-regulated RNAs in GD108-infected and Alpha-infected blood tissues. **C.** Upset plot displaying the intersection of the down-regulated RNAs in the blood tissues infected by different SARS-CoV-2 strains. **D.** The enriched GO terms for the RNA sets defined in (C). **E.** Heatmap showing the predicted immune cell proportion in the infected blood tissues. **F–I.** Intersection network of multiple tissues based on the ligand–receptor paires in GD108- (F), Alpha- (G), Beta- (H), and Delta-infected (I) blood tissues. The size of nodes represents the number of significantly induced ligands or receptors after SARS-CoV-2 infection in each organ; the direction of arrows indicates the signaling transduction from ligand to receptor between two organs; and the width of arrows indicates the number of significantly induced ligand–receptor pairs after infection between two organs. **J.** Genome browser showing the read abundance along *ITGAM* (ligand gene) and *SPON2* (receptor gene) in lung, blood, and right ventricle from control and infected rhesus macaques. **K.** qRT-PCR analysis showing the up-regulated expression of *SPON2* and *ITGAM* in lung/blood and blood/right ventricle tissues of Alpha-infected macaques, respectively. Three replicates were measured in each tissue sample. *, *P* < 0.05 and **, *P* < 0.01 (Student’s *t*-test).

Previous reports have revealed the active immune response in the peripheral blood mononuclear cells [32]. We hypothesized that the circulatory system for the body’s blood may contribute to the signaling transmission after infection. We first examined the up-regulated RNAs in the infected blood tissues (Figure 6A) and observed less than 40% of commonly up-regulated RNAs among all infected blood tissues (Figure S6A). Intriguingly, these commonly up-regulated RNAs were significantly enriched in Alpha-infected tissues (Figure 6B), but there was no difference in the commonly up-regulated RNAs between GD108-infected and Alpha-infected blood tissues (Figure S6B). The functional enrichment analysis showed that VOCs specifically induced the expression of RNAs involved in inflammatory and immune responses (Figure 6C). In addition, the down-regulated RNAs were specific among different strain infections (Figure S6C), and immune-related RNAs were specifically down-regulated in GD108 infection (Figure S6D). The ISGs were significantly down-regulated in GD108 infection and up-regulated in VOC infection, especially in Alpha infection (Figure 6D; Table S6). We also observed higher proportions of plasma cells, B-cells, and T-cells after infection based on immune cell prediction (Figure S6E), which is in line with a previous study [32]. Given the circulation characteristics of blood, we tried to illustrate the potential interactions between tissues across blood transmission using ligand–receptor pairs from CellTalkDB [33]. We observed a stronger signaling transmission among lung and right ventricle tissues with blood, especially in VOC infection (Figure S6F–I). For example, the ligand genes *S100A8* and *SPON2* were significantly up-regulated in Alpha-infected lung and blood tissues, and their receptor genes *ITGB2* and *ITGAM* were substantially expressed in the Alpha-infected blood and right ventricle (Figure 6E, Figure S6J), which were also validated by qRT-PCR (Figure 6F, Figure S6K). Those factors have been reported to be important in the regulation of inflammation and immune responses [34], [35], [36], [37]. Generally, VOC infection preferentially induced immune and inflammatory responses in blood tissues at 5 dpi, and blood might serve as the intermediate to promote an active immune response in the right ventricle from signaling of the infected lung tissues.

## Discussion

The innate immune system is an important defense against pathogen invasion [38]. Early innate immune-mediated inflammatory responses are crucial for host defense against viral infection [39]. However, if not controlled, an excessive inflammatory response can result in cytokine storms, and late inflammatory response may lead to tissue injury and multiple organ failure [40], [41]. SARS-CoV-2 can cause a severe disease characterized by prominent immunopathology resulting from a dysregulated innate immune response accompanied by a poor adaptive response [42]. Consistently, through analyzing the transcriptional disturbance of the host cells upon the infection with the viral strains of SARS-CoV-2, we observed that defense against virus and immune responses were significantly enhanced, and original strain of SARS-CoV-2 induced stronger innate immune responses than VOCs *in vitro* (Figure 1D). This may be related to the enhanced immune evasion of VOCs.

The mutations of SARS-CoV-2 affect virus phenotype, conferring a fitness advantage to the virus. These mutations can enhance viral replication, transmissibility, and immune escape [43], [44]. Previous studies have reported that both Alpha and Beta strains show increased transmissibility and immune escape [45], [46]. The Delta strain is significantly more transmissible than Alpha and Beta strains and the original strain [47] and contains enhanced immune evasion ability and replication fitness with a rapid increase in viral load in individuals [48]. Consistent with that, we observed that the Delta strain had the highest replication *in vitro* (Figure S2A) and the highest viral loads in rhesus monkey tissues (Figure 3E). In addition, the enhanced expression of innate immune antagonist sgRNAs, such as *N*, *Orf6*, and *Orf9b*, in Alpha strains also contributes to the transmissibility and immune evasion [26], [49]. For example, Orf6 of SARS-CoV-2 antagonizes the immune response by disrupting nucleocytoplasmic trafficking by binding to Rae1-Nup98 [50], [51], [52], and inhibition of STAT1 nuclear import [53]. Orf9b may inhibit IFN production through interacting with mitochondrial adapter TOM70 [26], [54]. Intriguingly, our data revealed that in addition to *N*, *Orf6*, and *Orf9b*, *Orf7ab* was significantly up-regulated in VOCs, especially in Delta strain (Figure 2). Previous studies have shown that Orf7a of SARS-CoV-2 reduces the antiviral effect of SERINC5 by affecting its expression [55] and can also degrade SNAP29 to initiate autophagy and limit autophagosome–lysosome fusion to promote viral replication [56]. In summary, viral mutations and enhanced sgRNA expression of VOCs increase the virus transmission and immune evasion.

Although COVID-19 is primarily considered a respiratory disease with high chances to progress to severe pneumonia, SARS-CoV-2 can affect multiple organ systems and induce organ injuries [20], [21]. Therefore, we observed SARS-CoV-2 original strain and VOC infection in multiple organs of rhesus macaques at 5 dpi (Figure 3A–D). Our results showed that SARS-CoV-2 original strain and its VOCs were able to infect these tissues and led to severe pathological changes in the lung and heart, while pathological changes in the brain were relatively weak at 5 dpi and may take a longer time to observe and evaluate. Neurological complications, such as the loss of smell and taste, headaches, fatigue, and impaired attention and memory, are manifestations of SARS-CoV-2 infection [20], [57], [58]. The nervous system dysfunction reported in COVID-19 patients may be caused by overstimulation of the immune system, and it is speculated that glial cells are strongly involved in the response to peripheral inflammation [59]. Overactivation of glial cells can lead to neuroinflammation and damage to the nervous system and eventually lead to dysfunction of neural circuits, with negative effects on cognitive and neuropsychiatric functions [60]. According to a previous report, inflammatory and immune response-related RNAs are specifically up-regulated in cerebral cortex at 7 dpi, which contributes to inflammatory encephalopathy [21]. However, we did not observe the significantly increased immune and inflammatory responses in cerebral cortex at 5 dpi. Our results suggest that inflammation and injury in the brain may occur relatively late. In addition, the cerebral cortex was more susceptible to SARS-CoV-2 GD108 and Alpha strains than the cerebellum given the transcriptome disturbance in these brain areas (Figure 4). Significantly, our data revealed that GD108 and Alpha strains of SARS-CoV-2 significantly disturbed the transcription in multiple tissues (Figure 3H); Delta strain slightly caused transcription changes with the highest viral loads, and Beta strain had relatively weak infectivity and disturbance in this study.

Most notably, right ventricle showed active immune and inflammatory responses at 5 dpi (Figure 5C). Cardiovascular symptoms, including palpitations, dyspnea, fatigue, and chest pain, are common in patients with SARS-CoV-2 [18], [60]. SARS-CoV-2-mediated cardiac injury has several distinct mechanisms [61], [62], including direct cell injury caused by SARS-CoV-2 [63], indirect injury from hypercoagulability and cytokine storm [64], and autoimmune-mediated injury triggered by the host response to the virus in susceptible individuals [65]. Autopsies performed in patients with SARS-CoV-2 infection revealed inflammatory cardiomyopathy [66]. Inflammatory cardiomyopathy is a common disease characterized by infiltration of immune cells in the heart. It can be complicated by heart failure with adverse consequences [67]. Macrophage polarization is closely related to the inflammatory response [31]. In this study, we found that M2 macrophages, which participate in predicted immune microenvironment in the right ventricle, were obviously increased after the infection by SARS-CoV-2 original strain and VOCs at 5 dpi (Figure 5E), and macrophage polarization-inducible factors were significantly up-regulated (Figure 5F, Figure S5E). These data further suggest that the heart develops an inflammatory response in the early stage of infection by SARS-CoV-2 original strain and its VOCs.

Previous studies have reported elevated levels of neutrophils, monocytes, chemokines, and inflammatory factors in peripheral blood of patients with COVID-19 [19], [68], [69], [70]. In this study, we also observed that immune and inflammatory responses were activated in blood after SARS-CoV-2 infection. Intriguingly, unlike the solid organs, SARS-CoV-2 VOCs specifically induced ISGs in blood tissues (Figure 6D), which could be partly explained by the specificity of blood tissues and the time sequence characteristics of individual tissues infected by SARS-CoV-2. Moreover, a stronger signaling transmission among lung and right ventricle tissues through blood was observed (Figure 6E), suggesting that blood as the circulatory system serves as the carrier for the substance exchange and assists signal transmission between tissues after infection. After SARS-CoV-2 infection, the signal transmission between organs relies on the systemic circulatory system, which may further enhance the inflammatory response.

In conclusion, the transcriptome-wide molecular changes of SARS-CoV-2 origianl strain and its VOCs both *in vitro* and *in vivo* established in this study provide insights into the pathogenesis of SARS-CoV-2 origianl strain and its VOCs in the early stage of infection, which will further deepen our understanding of COVID-19.

## Materials and methods

### Virus and cells

The SARS-CoV-2 original strain (GD108) and Beta variant (B.1.351) were from Guangdong Provincial Center for Disease Control and Prevention (CDC), Alpha variant (B.1.1.7) was from China CDC, and Delta variant (B.1.617.2) was from Chongqing CDC. African green monkey embryonic kidney MA104 cells (Catalog No. CL-0479, Procell, Wuhan, China) were cultured in Dulbecco’s modified eagle medium (Catalog No. C11995500BT, GIBCO, Grand Island, NY) supplemented with 10% fetal bovine serum (Catalog No. 10099141, GIBCO) and 1% penicillin–streptomycin (Catalog No. 15140-122, GIBCO) at 37°C and 5% CO_2_. The MA104 cells were separately infected with SARS-CoV-2 original strain (GD108) and its VOCs (Alpha, Beta, and Delta) at an multiplicity of infection (MOI) of 0.1 for 48 h, and MA104 cells treated with PBS (mock) for 48 h were used as a control.

### Animal experiments

In this study, rhesus macaques of 7–13 years old were used and housed at National Kunming High-level Biosafety Primate Research Center, China. All animal experiments conformed to the standards for use and care of laboratory animals. Rhesus macaques were infected with 1 × 10^7^ PFU of SARS-CoV-2 GD108 and its VOCs (Alpha, Beta, and Delta) at 0 dpi (*n* = 1), and one uninfected rhesus macaque was provided as a control. All macaques were euthanized at 5 dpi, and several parameters were measured, including body weight loss, pathological feature, and SARS-CoV-2 content in lung, right ventricle, cerebral cortex, and cerebellum.

### qRT-PCR

Viral loads in tissues were determined by measurement of viral *N* gene (forward: 5′-GACCCCAAAATCAGCGAAAT-3′; reverse: 5′-TCTGGTTACTGCCAGTTGAATCTG-3′; probe: 5′-FAM-ACCCCGCATTACGTTTGGTGGACC-BHQ1-3′). The mRNAs of *N*, *Orf6*, *Orf7ab*, and *Orf9b* were validated in MA104 cells using the comparative Ct (2^−ΔΔCt^) method, with *ACTIN* mRNA as the internal control. The primers used are listed in Table S7.

### Immunofluorescence staining assay

Immunofluorescence staining assay was performed according to standard protocols. Images were captured using a panoramic MIDI digital scanner (Catalog No. 3DHISTECH, Pannoramic MIDI, Budapest, Hungary). Anti-SARS-CoV-2 Nucleocapsid (NP; Catalog No. 40143-MM08, Sino Biological, China) were used in this study, and the secondary antibody was Alexa Fluor 488-conjugated Goat Anti-Mouse (Catalog No. A-11001, Invitrogen, Carlsbad, CA).

### Immunohistopathology

H&E staining was performed according to standard protocols. Images of H&E staining were captured using a panoramic MIDI digital scanner.

### RNA-seq library and sequencing

Total RNA was collected in the frozen tissues of rhesus macaques with TRIzol reagent (Catalog No. 15596018, ThermoFisher Scientific, Waltham, MA). VAHTS Universal V6 RNA-seq Library Prep Kit (Catalog No. NR604, Vazyme, Nanjing, China) was used to construct libraries with the obtained RiboMinus RNA following the manufacturer’s instructions. Paired-end sequencing was performed on the Illumina NovaSeq 6000 sequencing system with 150 bp read length.

### SARS-CoV-2 VOC genome assembly

Original strain (GD108) and VOCs including Alpha (B.1.1.7), Beta (B.1.351), and Delta (B.1.617.2) were sequenced by first-generation sequencing. Then SnapGene software was used for genome annotation by referring to SARS-CoV-2 Wuhan-Hu-1 complete genome (https://www.ncbi.nlm.nih.gov/nuccore/NC_045512).

### Quality control for RNA-seq data

Cutadapt (version 1.13) [71] was used to trim the adapters of raw sequencing reads, and Trimmomatic (version 0.36) [72] was applied to filter the short reads (< 35 bp) or the low-quality and ambiguous nucleotides. The quality of sequencing reads was evaluated by FastQC https://www.bioinformatics.babraham.ac.uk/projects/fastqc/.

### Viral RNA quantification from RNA-seq data

Filtered reads were mapped to the genomes of SARS-CoV-2 original stain and VOCs (GD108 accession: NC_045512.2; self-assembled genomes for VOCs are listed in Table S8) using BWA aligner (version 0.7.17) [73]. SARS-CoV-2 sgRNAs were characterized by the junction of the leader sequence with downstream sgRNA sequence. Mapped paired reads containing the 3′ end of the leader sequence “CTTTCGATCTCTTGTAGATCTGTTCTC” were extracted using SAMtools (version 1.9) [73]. Junction sites were labeled on the basis of the location of transcription regulatory sequences (TRSs) or other known sites with ±5 nt windows for each sgRNA (Table S9). BEDTools (version 2.26.0) [74] was applied to annotate the leader-containing reads to SARS-CoV-2 genomes and calculate the read counts for each junction site. The TRS of *Orf1ab* is the genomic junction site from 100 nt to 150 nt in the genomes of SARS-CoV-2 original strain and its VOCs. Junction read counts among SARS-CoV-2 original strain and its VOCs were normalized to have equal “genomic” reads and then calculated the ratios between VOCs and GD108. Linear regression of R program [75] was performed to evaluate the abundance of sgRNAs between VOCs and GD108.

### Host RNA-seq data processing and analysis

The clean reads were mapped to *Macaca mulatta* genome from Ensembl database (release 100) [76] using HISAT2 (version 2.0.2) [72] with default parameters. Host transcript abundances were estimated by the StringTie [77] program. Differentially expressed genes (DEGs) and reads per kilobase of exon model per million mapped reads (RPKM) were evaluated on the basis of read counts for exons of each transcript using featureCounts [78] and the fold change (FC) and significance were determined by the edgeR package of R program [79] with threshold of FC ≥ 2 and false discovery rate (FDR) < 0.05. UpSetR [80] was used to display the intersection of genes in different samples.

Clustering of tissue and cell samples was performed by principal component analysis (PCA) using the scaled RNA expression of fragments per kilobase million (FPKM). Gene clusters in the GD108-infected tissues at different time points were determined through 10,000 iterations of *K*-means algorithm [81] based on the gene expression levels. The Database for Annotation, Visualization and Integrated Discovery (DAVID) [82] was applied to annotate the Gene Ontology consortium [83] for different gene sets filtering by *P* < 0.05. RNA abundance was normalized by bamCoverage of BEDTools (version 2.26.0) [74] and displayed using the Integrative Genomics Viewer (IGV) [84].

CIBERSORT algorithm [30] was applied to predict the immune cell composition of different tissues on the basis of gene expression. Ligand–receptor pairs from CellTalkDB database [33] were applied to establish tissue communication and displayed by Cytoscape [85].

### Statistical analysis

One-way Analysis of Variance (ANOVA) was performed for multiple-group comparisons. Student’s *t*-test was used for two-group comparisons. GraphPad Prism 8 software (GraphPad, La Jolla, CA) was used to evaluate the significance for the qRT-PCR results, and the images were processed using Adobe Illustrator. Statically significant differences for the FCs of gene sets in different samples were evaluated by one-side unpaired Wilcoxon test. The hypergeometric distribution was applied to evaluate the significance of the overlapping RNAs between different samples. The asterisks were used to represent the statistical significance as * *P* < 0.05, ** *P* < 0.01, and *** *P* < 0.001.

## Ethical statement

All animal procedures were approved by the Institutional Animal Care and Use Committee of Institute of Medical Biology, Chinese Academy of Medical Sciences (Approval No. DWSP202002001), and performed in National Kunming High-level Biosafety Primate Research Center, China.

## Data availability

Raw RNA-seq data have been deposited in the Genome Sequence Archive [86] at the National Genomics Data Center, Beijing Institute of Genomics, Chinese Academy of Sciences / China National Center for Bioinformation (GSA: CRA009354), and are publicly accessible at https://ngdc.cncb.ac.cn/gsa.

## Competing interests

The authors have declared no competing interests.

## CRediT authorship contribution statement

**Tingfu Du:** Data curation, Formal analysis, Investigation, Methodology, Resources, Validation, Visualization, Writing – original draft. **Chunchun Gao:** Data curation, Formal analysis, Investigation, Methodology, Software, Validation, Visualization, Writing – original draft. **Shuaiyao Lu:** Methodology, Project administration, Resources, Writing – review & editing. **Qianlan Liu:** Investigation, Methodology, Writing – review & editing. **Yun Yang:** Methodology, Resources, Writing – review & editing. **Wenhai Yu:** Resources. **Wenjie Li:** Methodology. **Yong Qiao Sun:** Methodology. **Cong Tang:** Resources. **Junbin Wang:** Resources. **Jiahong Gao:** Methodology. **Yong Zhang:** Resources. **Fangyu Luo:** Resources. **Ying Yang:** Project administration, Resources, Supervision, Writing – review & editing. **Yun-Gui Yang:** Conceptualization, Funding acquisition, Project administration, Resources, Supervision, Writing – review & editing. **Xiaozhong Peng:** Conceptualization, Funding acquisition, Project administration, Resources, Supervision, Writing – review & editing. All authors have read and approved the final manuscript.

## Supplementary material

Supplementary Table S1 **The transcriptional expression characteristics of host cells before and post****infection by****SARS-CoV-2****original strain****and its VOC****s**

Supplementary Table S2 **The transcriptomic dysregulation of host cells post****infection by****SARS-CoV-2****original strain****and its VOC****s**

Supplementary Table S3 **The sgRNA read****counts of SARS-CoV-2****original strain****and its VOC****s**

Supplementary Table S4 **The body weight and temperature of rhesus macques post****infection by****SARS-CoV-2****original strain****and its VOC****s**

Supplementary Table S5 **The****transcriptome****s****of****tissues of rhesus macaques with or without infection by****SARS-CoV-2****original strain****and its VOC****s**

Supplementary Table S6 **The transcriptional changes****in****tissues****of rhesus macaques infected by****SARS-CoV-2****original strain****and its VOCs****at 5 dpi**

Supplementary Table S7 **Primers used in this study**

Supplementary Table S8 **SARS-CoV-2 VOC genome sequences**

Supplementary Table S9 **The TRS locations****in the genomes of****SARS-CoV-2****original strain****and its VOCs**
